# Supplementary material for: Virus-borne mini-CRISPR arrays are involved in interviral conflicts
Source: Nat Commun. 2019 Nov 15;10:5204. doi: 10.1038/s41467-019-13205-2 (PMC6858448; doi:10.1038/s41467-019-13205-2)
Supplement: Supplementary file 1 — Supplementary Information [file 41467_2019_13205_MOESM1_ESM.pdf]

SUPPLEMENTARY INFORMATION

Virus-borne mini-CRISPR arrays are involved in interviral conflicts

Sofia Medvedeva[1,2,3], Ying Liu[1], Eugene V. Koonin[4], Konstantin Severinov[2], David Prangishvili[1,5], Mart Krupovic[1]*

1 – Institut Pasteur, Department of Microbiology, 75015, Paris, France.
2 – Center of Life Sciences, Skolkovo Institute of Science and Technology, Skolkovo, Russia.
3 – Sorbonne Université, Collège doctoral, 75005, Paris, France.
4 – National Center for Biotechnology Information, National Library of Medicine, Bethesda, MD 20894, USA.
5 – Ivane Javakhishvili Tbilisi State University, Tbilisi, Georgia.

* - correspondence to
mart.krupovic@pasteur.fr

SUPPLEMENTARY TEXT

Temporal CRISPR spacer dynamics in the enrichment cultures

In the original environmental J15 sample, most spacers display similar abundances. However, after 10 days of cultivation, the community has visibly stratified into well-defined groups, each characterized by a specific frequency of spacers and likely representing a discrete strain. After 20 days of cultivation, the gap between the groups of high-abundance (≤10,000 coverage) and low-abundance (≤10 coverage) spacers increased for CRISPR-A, -B and -C. The moderate abundance spacers (10-1000 coverage) have largely disappeared, especially, in the case of CRISPR-A and CRISPR-C repeats, suggesting that the population became dominated by a handful of strains. The situation was different for the spacers associated with the CRISPR-D repeats: the 4 dominant groups of populations grew in abundance and spawned a small group of extremely abundant spacers (>10,000 coverage).

A different pattern was observed with the J14 sample, where we could compare the enrichment cultures of 10 and 20 days. Whereas the population structures for the CRISPR-B repeats followed the same course as in the J15 samples, the populations bearing the CRISPR-D repeats segregated to the high-abundance and low-abundance groups. By contrast, populations with the CRISPR-A repeats showed an increase in moderate abundance spacers (opposite to the situation in the J15 sample), whereas those with the CRISPR-C repeats evolved towards collapse, with the majority of the strains displaying very low abundance.

Assembly of viral contigs and CRISPR arrays

Using CRISPRome data, we were able to reconstruct contigs of up to 200 nucleotides (Supplementary Figure 3c), despite the fact that this approach was complicated by short (30-36 bp) spacer lengths and inherent absence of spacers from genomic regions devoid of the protospacer adjacent motifs (PAM). Following the *in silico* translation, matches to viral proteins were identified, as in the example shown in Supplementary Figure 3c, where the reconstructed contig encodes the structural protein VP2 of fuselloviruses. The reconstruction of the viral contigs from the CRISPRome data is conceptually similar to the reconstruction of plant virus genomes from small interfering RNA sequences.

Approximately 50% of our HTS sequencing reads include not solitary spacers but small fragments of CRISPR arrays with 2 or, less frequently, 3 spacers. The assembly of these fragments through identical spacers, theoretically, should allow reconstruction of longer CRISPR arrays. In practice, however, the spacer diversity of natural Sulfolobales population can only be represented as a graph (Supplementary Figure 8), which, in some cases, cannot be resolved into separate CRISPR arrays, due to intrinsic variations, such as deletion of spacers in the trailer end of CRISPR arrays or acquisition of new spacers at the leader end. To overcome this problem, we introduce the eccentricity metrics. The eccentricity of a spacer is the length of the longest CRISPR array, which can be reconstructed with this spacer (Supplementary Figure 7b). The longest CRISPR arrays (the maximal eccentricity) were 131, 66, 139 and 119 for spacers associated with the CRISPR-A, -B, -C and -D repeats, respectively. These length estimates agree with the average lengths of arrays in sequenced Sulfolobales isolates. The eccentricity > 3 was observed for 38% of all spacers associated with the CRISPR-A repeats and 98% of spacers with abundances > 100. Each Sulfolobales genome usually contains more than one CRISPR array with the same CRISPR repeat sequences. We observed groups of spacers from 3 independent graph components with linearly correlated frequencies in two samples (Supplementary Figure 9), which is consistent with them being sequenced from the same genome.

Detection of integrated MGE by spacer matching

Archaeal viruses and plasmids are known to integrate into the genomes of their hosts. For many of these integrated MGE, closely related extrachromosomal relatives are not known, making their identification cumbersome. Mapping the CRISPR spacers against the Sulfolobales chromosomes provides an efficient approach to identify integrated MGEs, both related to known plasmids and viruses as well as novel and even deteriorating ones. A threshold of 3 protospacers per kb of genomic DNA was found to be a reliable predictor for the presence of integrated MGEs. Using this approach, we predicted 11 MGEs integrated in 9 Sulfolobales genomes and subsequently validated the precise integration sites for all but one element (Supplementary Figure 3a; Supplementary table 4). These integrated MGE included 2 STIV-like proviruses, 7 integrated pNOB-like conjugative plasmids and 2 cryptic integrated elements. Some of the elements were apparently inactivated by transposon insertions and are unlikely to be mobile. Collectively, these integrated MGEs are targeted by 336 distinct spacers from our collection.

SUPPLEMENTARY FIGURES

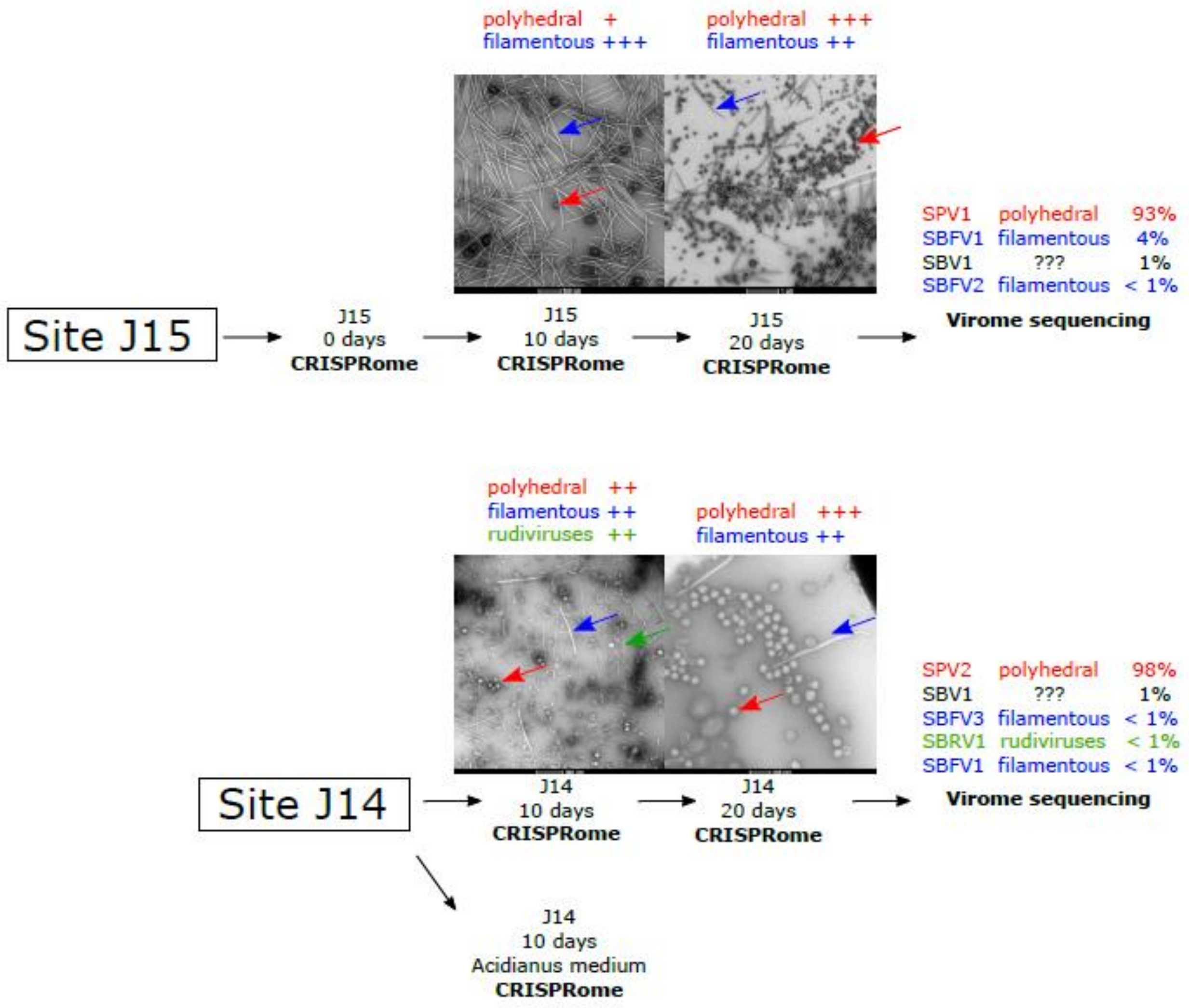

Supplementary Figure 1. Description of samples. Schematic representation of analysed samples. When availiable, images of virus diversity in enrichments are shown. Viruses belonged to different families are highlighted with arrows (polyhedral – red, filamentous – blue, rudiviruses – green).

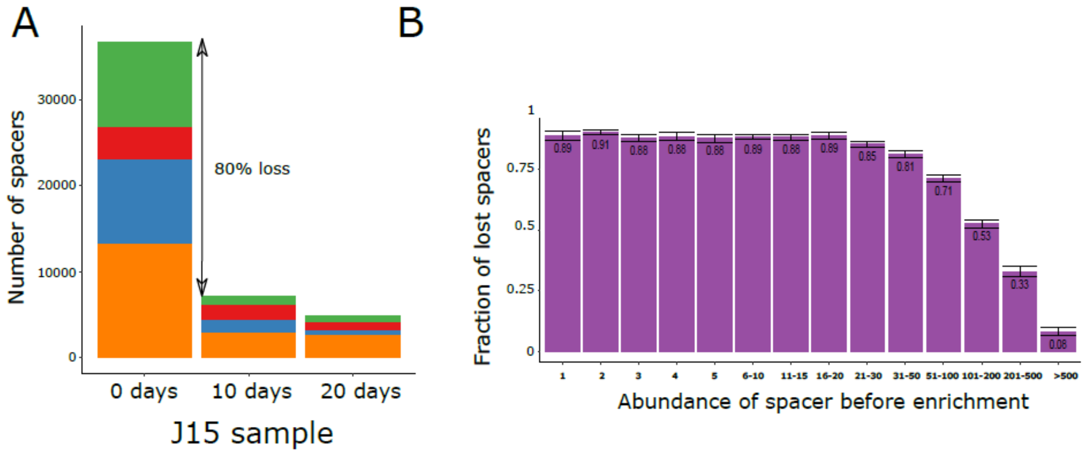

Supplementary Figure 2. Loss of spacers during cultivation. a. Number of spacers associated with four CRISPR consensuses are shown as a barplot for J15 sample. **b.** Fraction of spacers lost in all enrichment cultures is shown for groups of spacers with different abundances. Error bars show confidence intervals for the proportion.

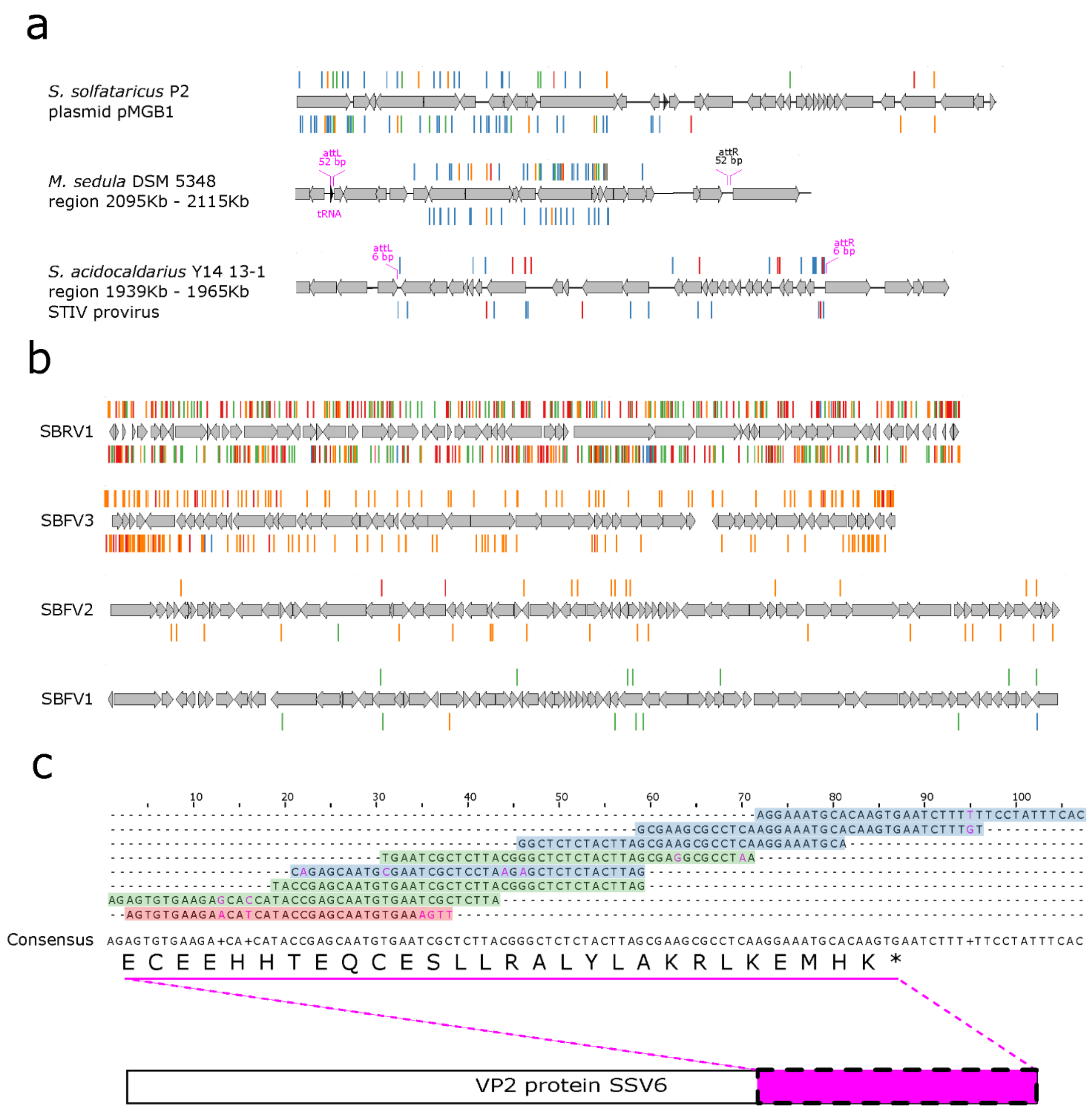

Supplementary Figure 3. a. Maps of several Sulfolobales integrated and extrachromosomal elements targeted by the Beppu CRISPR spacers. Protospacers are shown as thin bars above and below the genes (represented by grey arrows) depending on the targeted strand. The color of spacer bars corresponds to CRISPR consensuses. Identified attachment sites (attL and attR) for the integrated elements are shown in pink. The visualization is created by R package Gviz (https://bioconductor.org/packages/release/bioc/html/Gviz.html). **b.** Genome maps of several Sulfolobales viruses, targeted by Beppu CRISPR spacers. **c.** An example of viral contig reconstruction by overlapping spacer sequences. The color of spacers in alignment corresponds to CRISPR consensus. Not conserved positions in the alignment are highlighted by pink color. Consensus nucleotide sequence and protein translation are shown below the alignment.

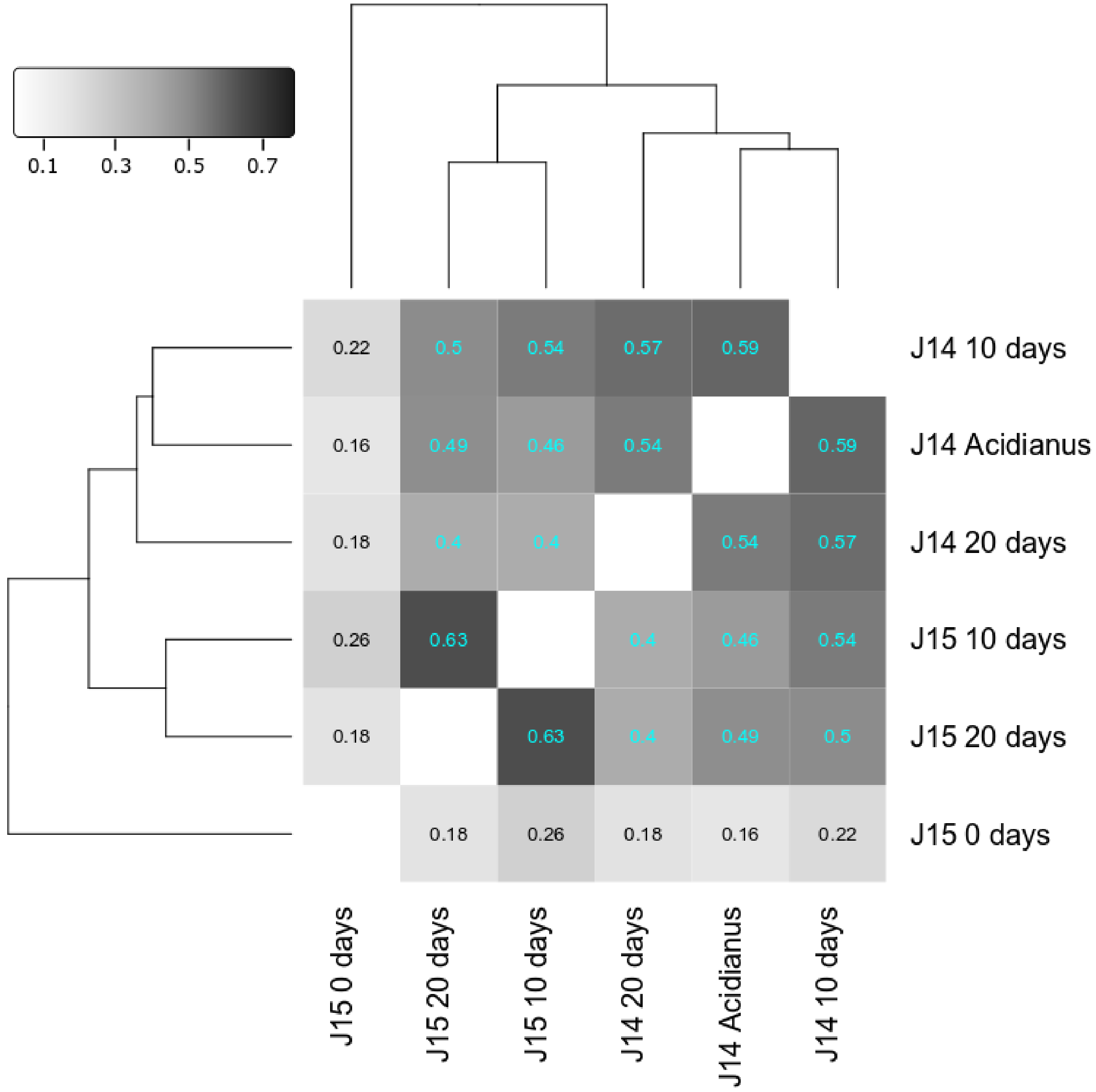

Supplementary Figure 4. Heatmap of fraction of spacers shared between samples. Then fraction of shared spacers for two samples is calculated as $2a/(2a + b + c)$, where a is a number of spacers shared between two samples, whereas b and c – number of unique spacers in each sample.

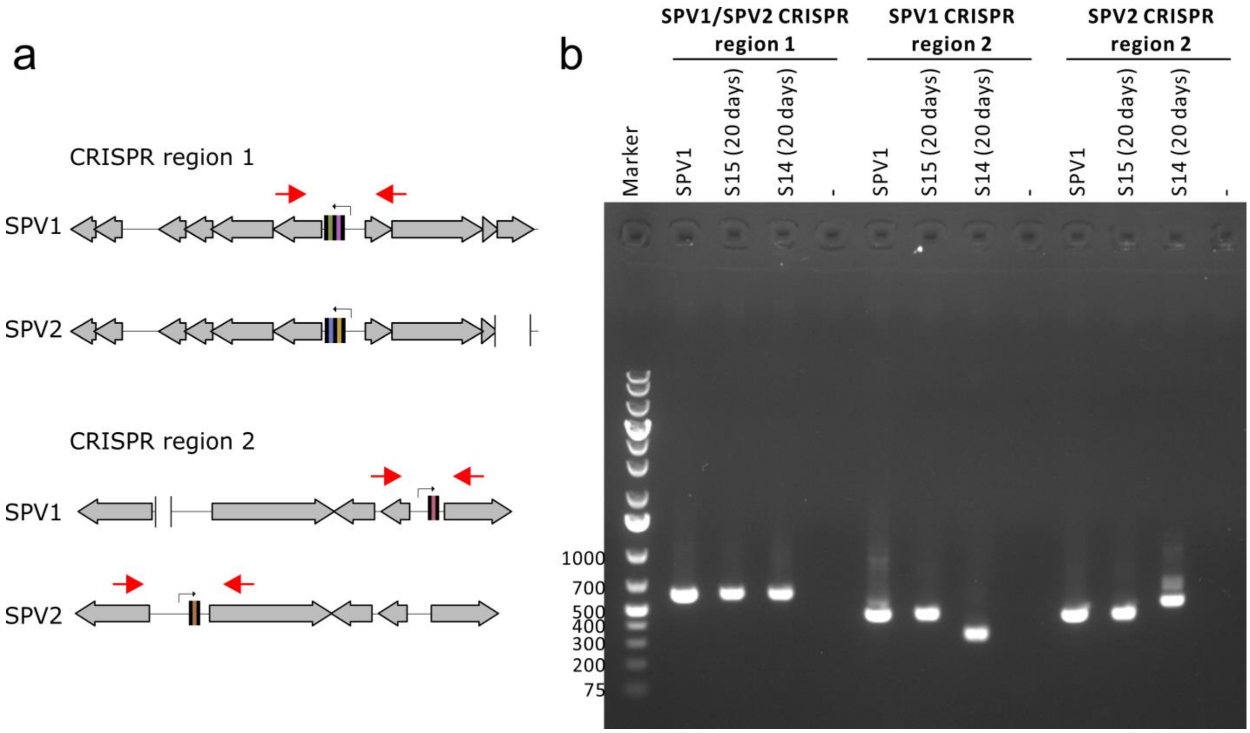

Supplementary Figure 5. Verification of mini-CRISPR regions of SPV1 and SPV2. **a**. The scheme showing the location of primer pairs targeting SPV1/SPV2 CRISPR region 1, SPV1 CRISPR region 2 and SPV2 CRISPR region 2. Note that CRISPR region 1 can be amplified with the same primer pair and produces fragments of the same length. Locations where the primers anneal are indicated with red arrows. **b**. PCR performed with the three pairs of primers using DNA isolated from SPV1 virions as well as cell fractions of enrichment cultures S15 (20 days) and S14 (20 days) as templates. "-" indicates the negative control with H_2O as a template.

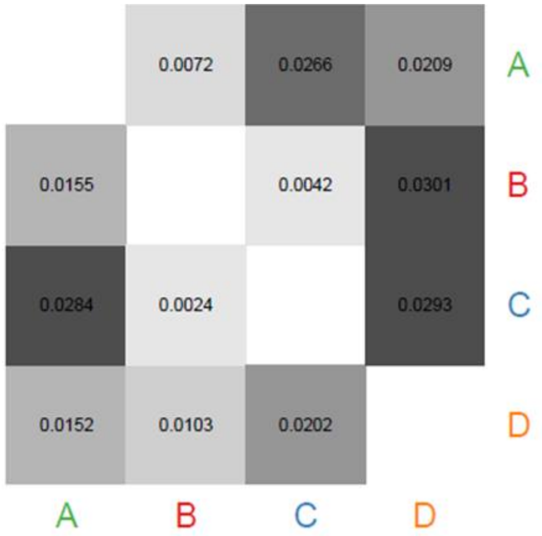

Supplementary Figure 6. Intersection between spacer sets of different CRISPR repeats. The fraction of spacers from the CRISPR repeat type in a row matching with CRISPR repeat type in the column is indicated.

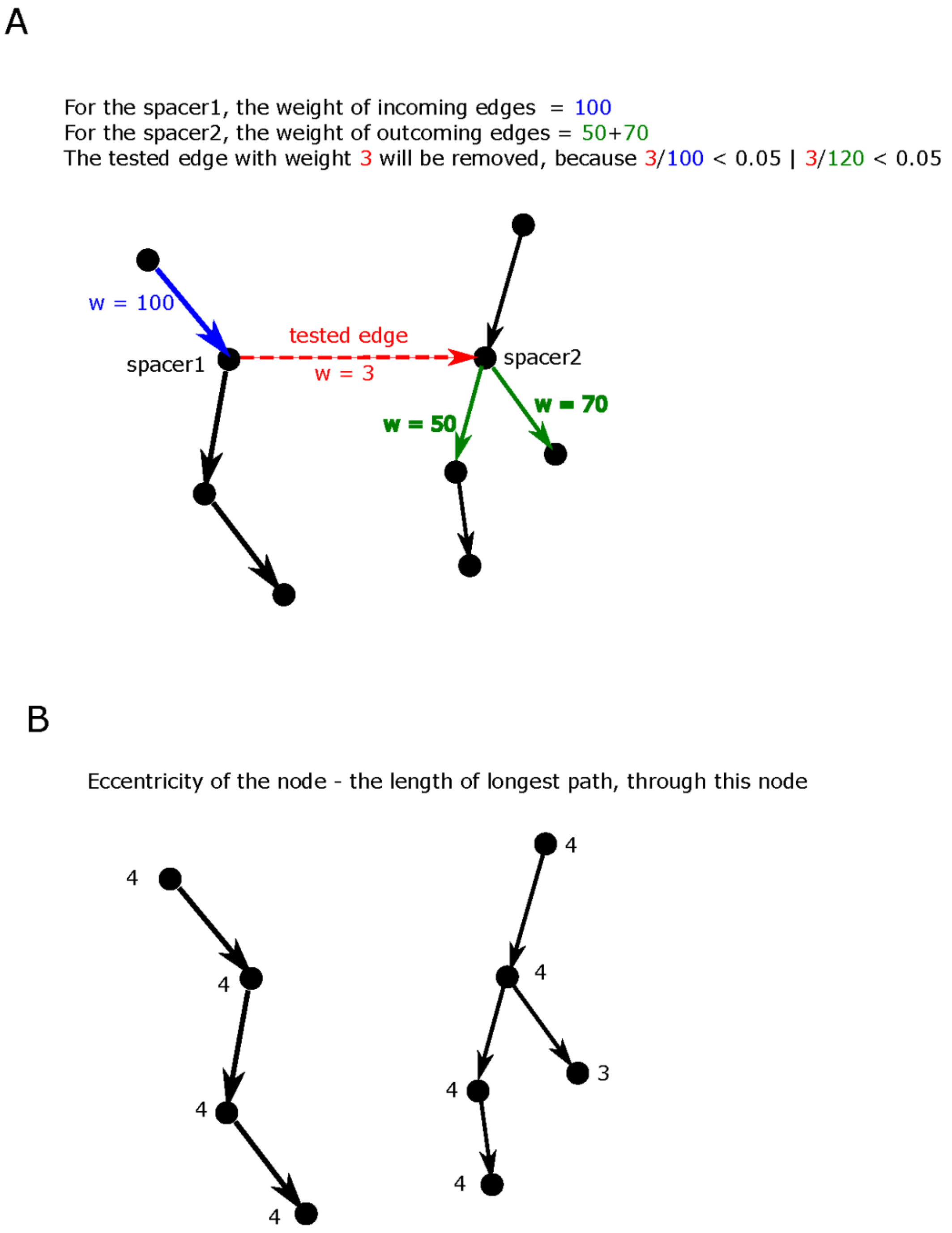

Supplementary Figure 7. Methods of reconstruction of long CRISPR arrays. **a.** Filtration of CRISPR array graph, by removing low abundant edges. **b.** Example of eccentricity calculation

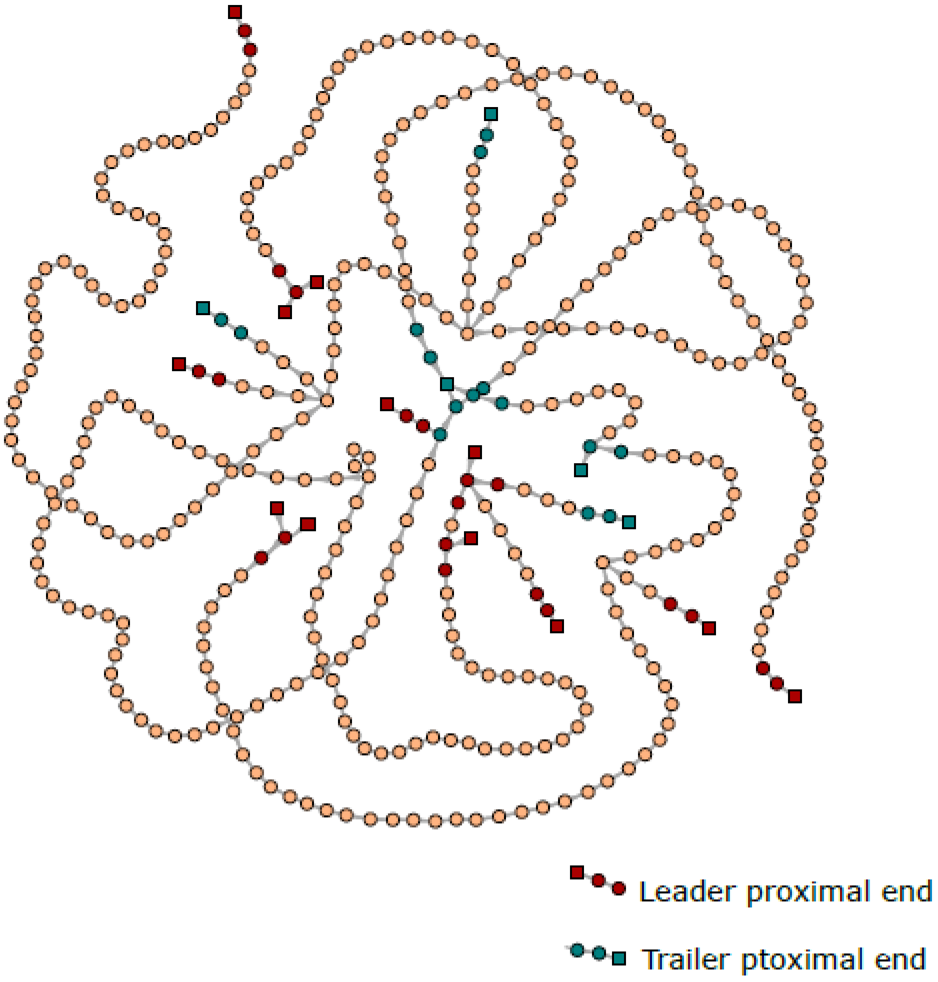

Supplementary Figure 8. An example of reconstructed CRISPR array graph. Three spacers in leader-proximal or trailer-proximal ends are highlighted with red and green respectively.

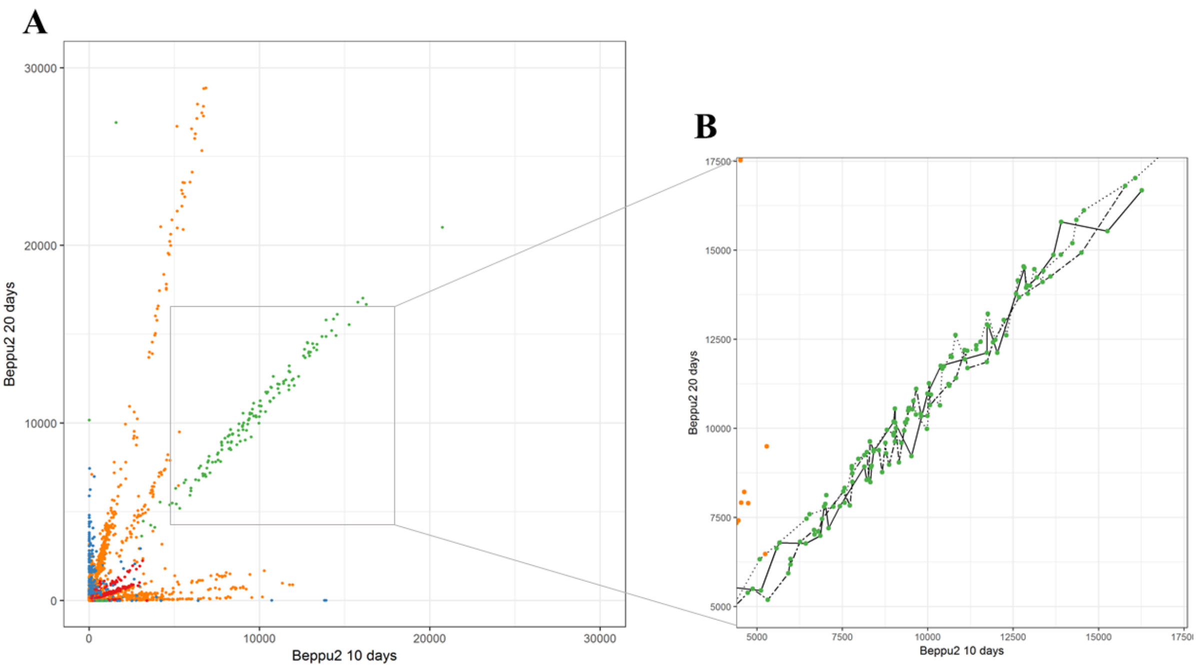

Supplementary Figure 9. Spacers with linearly changed frequencies in 10 and 20 days enrichments of J14 sample. **A.** All spacers of J14 sample. **B.** Zoom into a region with spacer abundances 5000-17500. Dashed, dotted and solid lines represent three independent components of CRISPR arrays graph.

SUPPLEMENTARY TABLES

Supplementary table 1. Distribution of CRISPR consensuses in Sulfolobales genomes.

CRISPR consensus	Sulfolobales genomes
A	*Metallosphaera , Acidianus, Sulfolobus*
B	*Acidianus, Sulfolobus*
C	*Metallosphaera*
D	*Metallosphaera, Acidianus, Sulfolobus*

Supplementary table 2. Diversity and coverage estimations for Beppu spacer sets.

Sample	CRISPR spacers (total)	Clusters	Good's criterion	S-Chao	alpha diversity (Shannon)
J15 – 0 days	2 971 721	33 991	0.91	36 068 (+-97)	9.18
J15 – 10 days	5 166 123	6 155	0.88	9 431 (+-439)	7.12
J15 – 20 days	4 787 974	4 462	0.76	5 868 (+-115)	5.80
J14 – 10 days	4 129 915	6 825	0.91	8 756 (+-251)	7.16
J14 – 20 days	4 540 454	4 585	0.84	6 573 (+-229)	6.56
J14 "Acidianus" – 10 days	3 234 790	4 020	0.87	4 332 (+-35)	5.96

Supplementary table 3. Primers sequences for amplification of CRISPR arrays of Sulfolobales and SPV1/SPV2.

Repeat	Forward primer (5'-3')	Reverse primer (5'-3')
G1	TCGTCGGCAGCGTCAGATGTGTATAAGAGACAGCTTTTCTCTTATGAGACTAGTAC	GTCTCGTGGGCTCGGAGATGTGTATAAGAGACAGCTAGTCTCATAAGAGAAAAGTAAT
A	TCGTCGGCAGCGTCAGATGTGTATAAGAGACAGTAATCTACTATAGARTTGAAAG	GTCTCGTGGGCTCGGAGATGTGTATAAGAGACAGTTCAAYTCTATAGTAGATTADC
B	TCGTCGGCAGCGTCAGATGTGTATAAGAGACAGAAYAACGAMAAGAAACTAAAAC	GTCTCGTGGGCTCGGAGATGTGTATAAGAGACAGTTTAGTTTCTTKTCGTTRTTAC
C1	TCGTCGGCAGCGTCAGATGTGTATAAGAGACAGAACCCTCAAAGGATCACTACAA	GTCTCGTGGGCTCGGAGATGTGTATAAGAGACAGGTGATCCTTTGAGGGTTTGAAAC
C2	TCGTCGGCAGCGTCAGATGTGTATAAGAGACAGGWGATCCTTMGAGGGTTTGAAAC	GTCTCGTGGGCTCGGAGATGTGTATAAGAGACAGACCCTCKAAGGATCWCTACAAAC
D	TCGTCGGCAGCGTCAGATGTGTATAAGAGACAGTKAATCCYAAAAGGRATTGAAAG	GTCTCGTGGGCTCGGAGATGTGTATAAGAGACAGTTCAATYCCTTTTRGGATTMATC
SPV1/2 region 1	TAACTCCATTGCCCTTACCG	AGACCTCCTACAATCCTCCTCA
SPV1 region 2	ACTCTGTGTACCCGGGGTTT	ATGCCGTAAGCCTTGTCTTG
SPV2 region 2	ACCCGTTTGCCTTTACACTG	TCAATATACTGCGTTTGTTTTCC

Adaptor sequences are underlined.

Supplementary table 4. Identified integrated elements in Sulfolobales genomes.

Accession number	Strain	Start	End	Size, bp	# spacers	Element type
CP000682.1	*M. sedula* DSM 5348	2096383	2112116	15734	58	cryptic (inactivated)
BA000023.2	*S. tokodaii* str. 7	~262600	~274200	11500	20	cryptic (inactivated)
BA000023.2	*S. tokodaii* str. 7	1310850	1355729	44880	93	conjugative
CP001399.1	*S. islandicus* L.S.2.15	1858852	1900333	41482	23	conjugative (inactivated)
CP001401.1	*S. island*icus M.16.27	1437439	1481760	44322	20	conjugative
CP001402.1	*S. islandicus* M.16.4	1474356	1512307	37952	25	conjugative
CP001403.1	*S. islandicus* Y.G.57.14	1465198	1505472	40275	23	conjugative
CP001731.1	*S. island*icus L.D.8.5	1323689	1390124	66436	27	conjugative
CP020362.1	*S. acidocaldarius* Y14 16-22	395173	437039	41867	20	conjugative (inactivated)
CP020362.1	*S. acidocaldarius* Y14 16-22	1991521	2008456	16936	27	provirus (STIV-like)
CP020363.1	*S. acidocaldarius* Y14 13-1	1943014	1959949	16936	27	provirus (STIV-like)
