# Supplementary material for: Virus-borne mini-CRISPR arrays are involved in interviral conflicts
Source: Nat Commun. 2019 Nov 15;10:5204. doi: 10.1038/s41467-019-13205-2 (PMC6858448; doi:10.1038/s41467-019-13205-2)
Supplement: Supplementary file 3 — Description of Additional Supplementary Files [file 41467_2019_13205_MOESM3_ESM.docx]

**Description of Supplementary Files**

**File Name:** Supplementary Data 1

**Description:** Collection of 40704 unique spacer sequences obtained in the study. The type of associated CRISPR repeat and abundance of each spacer are included into header lines.

**File Name:** Supplementary Data 2

**Description:** Description of 6 spacers found in SPV1 and SPV2 mini-CRISPR arrays.
